# Developing Mobile Health Interventions With Implementation in Mind: Application of the Multiphase Optimization Strategy (MOST) Preparation Phase to Diabetes Prevention Programming

**DOI:** 10.2196/36143

**Published:** 2022-04-26

**Authors:** Megan MacPherson, Kohle Merry, Sean Locke, Mary Jung

**Affiliations:** 1 School of Health and Exercise Sciences University of British Columbia Kelowna, BC Canada; 2 School of Rehabilitation Sciences University of British Columbia Vancouver, BC Canada; 3 Faculty of Kinesiology Brock University St Catherines, ON Canada

**Keywords:** text messaging, prediabetic state, telemedicine, telecommunications, exercise, diet, preventive medicine, mHealth, intervention development, behavior change, mobile phone

## Abstract

With thousands of mobile health (mHealth) solutions on the market, patients and health care providers struggle to identify which solution to use and prescribe. The lack of evidence-based mHealth solutions may be because of limited research on intervention development and the continued use of traditional research methods for mHealth evaluation. The Multiphase Optimization Strategy (MOST) is a framework that aids in developing interventions that produce the best-expected outcomes (ie, effectiveness), given constraints imposed on affordability, scalability, and efficiency (also known as achieving intervention EASE). The *preparation phase* of the MOST highlights the importance of formative intervention development—a stage often overlooked and rarely published. The aim of the preparation phase of the MOST is to identify candidate intervention components, create a conceptual model, and define the optimization objective. Although the MOST sets these 3 targets, no guidance is provided on how to conduct quality research within the preparation phase and what specific steps can be taken to identify potential intervention components, develop the conceptual model, and achieve intervention EASE with the implementation context in mind. To advance the applicability of the MOST within the field of implementation science, this study provides an account of the methods used to develop an mHealth intervention using the MOST. Specifically, we provide an example of how to achieve the goals of the preparation phase by outlining the formative development of an mHealth-prompting intervention within a diabetes prevention program. In addition, recommendations are proposed for future researchers to consider when conducting formative research on mHealth interventions with implementation in mind. Given its considerable reach, mHealth has the potential to positively affect public health by decreasing implementation costs and improving accessibility. The MOST is well-suited for the efficient development and optimization of mHealth interventions. By using an implementation-focused lens and outlining the steps in developing an mHealth intervention using the preparation phase of the MOST, this study may guide future intervention developers toward maximizing the impact of mHealth outside academia.

## Introduction

### Background

Without effective, affordable, scalable, and efficient interventions to prevent or delay the onset of type 2 diabetes (T2D), >1 billion people will have or be at risk of developing diabetes by 2045, with an estimated annual global health expenditure of US $845 billion [1]. The efficacy of dietary and physical activity behavior change programs on T2D incidence has been shown in several large-scale randomized trials [2-5]. Specifically, these diabetes prevention programs (DPPs) have resulted in reductions in T2D incidence by 28% to 58%. That said, the protective effects of these behavior changes are largest during intensive DPPs, and the relative risk reduction tends to wane over time [6]. This is likely because of the decreased adherence to dietary and physical activity recommendations. For example, the Finnish DPP found that those who engaged in more physical activity during the 4-year follow-up saw larger reductions in T2D risk; however, 38% of participants in the behaviour change intervention group and 54% of participants in the control group did not meet the physical activity recommendations of 150 minutes of moderate to vigorous physical activity during this time [7].

Although the results from DPPs are promising, there is potential for time- and cost-efficient maintenance interventions to improve adherence to long-term behavior changes. Given the wide availability and accepted use of mobile technologies in daily life, mobile health (*mHealth*; defined as health services that are delivered by mobile devices) offers the potential to improve DPP service delivery and patient outcomes by providing the opportunity for patients to leverage the technologies they are already using to learn about their conditions, monitor their health-related behaviors and outcomes, and actively participate in their own health care [8]. Despite this promise, the rapidly evolving landscape of health technologies has resulted in a dearth of evidence-based mHealth interventions used in clinical practice [9]. Furthermore, mHealth tool and service design are often driven by the technology sector (not the health care industry), which prioritizes the rapid development of technologies to reduce time to market, often at the cost of the rigorous development and evaluation processes associated with traditional medical device and intervention design [9,10].

The challenge of developing and translating effective and scalable mHealth interventions into practice to improve public health, preventive medicine, and health communication has been well documented [11,12]. In fact, it has been noted that research findings can take up to 17 years to be integrated into clinical practice [13]. Even more disheartening is the fact that 50% of clinical innovations never reach widespread clinical use [14], and 80% of medical research dollars do not make a public health impact and result in *research waste* [15].

At this pace, technological advances will perpetually outpace research. Furthermore, when technologies are designed for research purposes without clinical implementation in mind, they may need significant modifications to be applicable for use in practice, resulting in increased cost and time—barriers that hinder the delivery of potentially meaningful mHealth programs to those in need [16]. Establishing the effectiveness of an mHealth innovation is insufficient to ensure its uptake in routine practice [17]. The question then remains: how can rigorous, evidence-based mHealth interventions be developed for clinical practice in a time-sensitive manner?

A potential method is to use methodological frameworks to aid in navigating the process from mHealth development to its implementation. This paper provides an example and recommendations for how the *Multiphase Optimization Strategy* (MOST) can be used to develop mHealth interventions and how the implementation context can drive decision-making throughout. Implementation science focuses not on the impact of an mHealth innovation but rather on the factors that influence the adoption of that innovation into routine use [17]. Implementation science has been defined as “the scientific study of methods to promote the systematic uptake of research findings and other evidence-based practice into routine practice and, hence, to improve the quality and effectiveness of health services” [18]. By using the MOST and integrating an implementation-focused lens early in formative mHealth development, mHealth interventions can be developed in a way that will ensure that they are not only effective but also immediately scalable [19].

### The MOST Framework

#### Overview

mHealth interventions are generally a combination of intervention components packaged together and offered to participants (eg, a weight-loss intervention delivered via a mobile phone app that includes diet logging, push notifications providing feedback on the foods eaten, and additional video conferencing with a dietitian to develop and refine a meal plan). Although traditional behavioral trial designs (eg, randomized controlled trials [RCTs]) can provide evidence of whether an mHealth intervention package is better than a control, such designs cannot determine which components contribute most to an outcome and the presence of potentially inert components resulting in inefficient mHealth interventions with unnecessary or possibly detrimental components. The MOST provides a structured, 3-phase, engineering-inspired framework in which intervention development and optimization precede an RCT (or other methods of evaluation). A potential use of the MOST within mHealth is the ability to develop resource-efficient mHealth interventions that include only active components delivered at an optimal dose. Using this strategy, interventions can be developed so that they are not only effective but also efficient, economical, and scalable—all attributes that are necessary for the development of mHealth interventions with implementation in mind. The 3 phases of the MOST include *preparation*, *optimization*, and *evaluation*. The aim of the preparation phase of the MOST is to identify candidate intervention components, create a conceptual model mapping target behaviors to outcomes, and define the optimization objective. Next, optimization trials are conducted to identify the *optimized* intervention, given certain implementation constraints. Finally, optimization may be followed by an evaluation phase, in which the optimized intervention package is evaluated (often in an RCT). This study focuses on the preparation phase of the MOST. For more information pertaining to the optimization and evaluation phases see Collins [20].

#### Preparation Phase

*Intervention components* are defined as any feature that can be manipulated for study by turning it on or off or setting them to be high or low [20] and can include program content (eg, behavior change techniques [BCTs]), fidelity components (eg, provision of additional education to those moderating group chats within an mHealth app to ensure consistent tone and language across all moderators), engagement components (eg, providing badges and rewards to encourage adherence), and delivery components (eg, timing and frequency of contact with a participant).

The *conceptual model* outlines how candidate components are expected to theoretically influence short- and long-term outcomes of interest. Without this forethought, intervention components may be chosen as “it sounded like a good idea at the time,” which can limit the utility of an intervention. By creating a conceptual model that identifies the proposed mechanisms of action, the MOST encourages well-thought-out mHealth interventions that can be built on and generalized beyond a single program of research.

The *optimization objective* describes how intervention EASE will be achieved by balancing an intervention's *Effectiveness* against its *Affordability*, *Scalability*, and *Efficiency*. By considering implementation constraints at the onset, the MOST has the potential to create digital interventions that expedite the translation of evidence-based mHealth innovations into clinical practice, thereby maximizing their impact on public health [21].

There is growing interest in applying the preparation phase of the MOST in implementation science [19] and digital behavior change interventions [21], and recent guidelines have been published to ensure transparent and consistent reporting within the preparation phase [22]. Although the MOST outlines these 3 targets within the preparation phase (ie, identification of intervention components and the optimization objective and creation of the conceptual model), little guidance is provided on *how* to conduct quality research within the preparation phase of the MOST, what steps should be taken to decide on what technologies should be used in the intervention, and how to systematically identify potential intervention components, develop the conceptual model, and achieve intervention EASE with the implementation context in mind. This paper provides an example of this by outlining the formative development of an mHealth-prompting intervention to be used within the Small Steps for Big Changes (SSBC) DPP with the aim of improving long-term behavior change adherence.

### The SSBC Program

SSBC is an evidence-based, brief diet and exercise counseling program that aims to reduce individuals’ risk of developing T2D [23]. SSBC comprises two phases: (1) training and (2) follow-up. The training phase includes 6 one-on-one dietary and exercise counseling sessions and supervised exercise sessions facilitated by a YMCA coach over a span of 3 to 4 weeks. The follow-up phase includes check-ins and measurements for months following program completion. Participants are asked to continue using the strategies they learned in the program to engage in diet and exercise behaviors without any continued coaching support.

The SSBC program has been shown to improve both cardiorespiratory fitness and cardiometabolic risk factors in a university laboratory-based study [23] and has been successfully transitioned from the laboratory into the community [24]. In the laboratory-based randomized trial, accelerometer-measured physical activity significantly increased after program completion when compared with that of baseline measures [23]. Effectiveness data for the community-based program on behavior change outcomes were evaluated using self-report measures 6 months after program completion and showed continued participant engagement in diet and exercise behaviors [24].

Participants in both the laboratory-based and community-based trials, which were conducted in Kelowna, British Columbia, Canada (the province’s third-largest metropolitan area, with an approximate population of 152,000) [25], were provided with mHealth platforms in which they were asked to continue to self-monitor their exercise behaviors during both the training and follow-up phases. In the laboratory-based trial, participants were asked to digitally self-monitor daily whether they exercised, took a day off (participants were prescribed 4 days off per week), or did not exercise (if they exceeded their number of days off) on an mHealth platform that is no longer available (Motivation Engine). During the laboratory-based trial, participants were provided with additional BCTs, including mHealth *prompts* (BCT 7.1) sent via push notifications and *rewards* (BCT 10.6), for continued self-monitoring. In the community-based trial, participants self-monitored their activity on a different mHealth platform (HealthWatch360), in which they were asked to log only the days in which they exercised. On this platform, no additional BCTs were used to encourage continued engagement.

Following completion of SSBC, a focus group was conducted to allow SSBC participants to share the challenges they faced while making diet and physical activity changes after the intensive SSBC training phase [26]. Key recommendations from this work include the creation of platforms to communicate information about prediabetes and receive ongoing support from their coaches. Further qualitative work (4 semistructured interviews; before and after training and at 3 and 12 months after completion of the training phase) found that a key facilitator of maintaining long-term behavior changes was the use of mHealth technologies [27]. As such, this study aimed to assess the utility of mHealth prompts within SSBC and develop an mHealth-prompting intervention to provide continued support to clients in their behavior change journey.

### Recommendations to Develop mHealth-Prompting Interventions Using the Preparation Phase of the MOST

#### Overview

The following sections present a high-level overview of the steps taken to develop an mHealth-prompting intervention to be implemented within the follow-up phase of SSBC, situated within the preparation phase of the MOST. The intended focus of this paper is on the *process of decision-making* involved in choosing possible components and component levels and the development of content within an mHealth-prompting intervention and not the *outcome* of this research. This work was written in accordance with the MOST PREP-REP (Preparation Reporting) checklist by Landoll et al [22] (Multimedia Appendix 1).

On the basis of lessons learned through these steps, coupled with key design considerations posited within the *Person-Based Approach* [28], *agile innovation* [29], and *user-centered design* [10], recommendations are provided for future mHealth developers to promote implementation considerations throughout the preparation phase of the MOST. Specifically, recommendations drawn from these 3 development strategies include a focus on end users throughout, integration of existing research or rapid intervention prototyping, and iterative design and testing [10,28,29]. This presents a single use case of the preparation phase of the MOST; as such, these recommendations may not be generalizable to all applications of the MOST.

#### Step 1: Select Appropriate mHealth Technology

##### Recommendation

Although mHealth is wide reaching, the uptake and availability of certain technologies are not equal across all populations. Therefore, researchers *must* consider the contextual factors of the target population before deciding which mHealth technologies will be used or developed to promote feasible implementation [28]. The target population should encompass all potential stakeholders (ie, those using the technology both directly or indirectly) and contextual factors derived from the physical environment or clinical workflow in which the technology is to be implemented [16]. For example, if a researcher develops an mHealth intervention that is inaccessible to the target population in the real-world context but then provides the technology to research participants to prove its efficacy, this mHealth intervention is only effective within the sphere of academia. Such research findings may still have implications in policy (eg, informing health care coverage to include novel technologies) but are unlikely to be translated quickly into practice to improve public health [17].

##### SSBC Example

As SSBC begins to scale up across Canada, mHealth technologies provide an opportunity to improve long-term behavior change adherence while providing participants with continued support from program providers in an accessible manner. Although much of the mHealth research and development has focused on smartphones and associated apps, such technologies are less likely to reach rural Canadian populations (ie, Canadians at increased risk for developing T2D) [30]. However, cellular phone ownership and SMS text messaging rates are increasing, with 99.7% of Canadians covered by mobile phone networks [30]. To date, SSBC has been implemented within Kelowna, a metropolitan area. However, as SSBC expands to more rural communities, mHealth prompts sent via SMS text messaging have the capacity to reach and engage with the largest number of potential SSBC participants compared with the mHealth prompts sent via push notifications (through an mHealth platform) in the laboratory-based trial.

#### Step 2: Assess Potential Impact of mHealth Technology Within the Target Context

##### Recommendation

Before investing resources in developing an mHealth intervention, researchers should first evaluate the intervention’s potential to influence behaviors within a given context [16]. Common pitfalls in formative development include spending too much or too little time on this phase [16]. The use of existing data, conducting research using methods that prioritize speed and simplicity (eg, rapid prototyping and innovation sprints), conducting or consulting reviews of the literature, and conducting qualitative research with the target end users can aid in the evaluation of the potential utility of a given technology for the population of interest [29]. Furthermore, agile innovation suggests that creating a clear termination and evaluation plan a priori may avoid continuing to invest resources in an intervention that does not suit the intended users or target context [16].

##### SSBC Example: Secondary Analysis

To identify the potential utility of mHealth prompts sent via SMS text messaging as SSBC expands to more rural areas, exploratory analyses were conducted to identify how long participants self-reported that they exercised on their respective mHealth platforms during the laboratory-based trial (when they received mHealth prompts sent via push notifications) and community-based trial (when they received no additional BCTs to increase engagement) [31]. We found that 83% of participants in the laboratory-based trial (ie, those who received prompts) self-reported exercise on their mHealth platform for an average of 82 days, whereas only 34% of participants in the community-based trial (those who did not receive prompts) self-reported exercise on their mHealth platform for an average of 43 days.

Following this, a more in-depth analysis of the laboratory-based trial was conducted to determine the acute impact a prompt may have on self-monitoring (ie, any day in which they logged *yes*, *no*, or *day off*) and self-reported exercise (ie, only the days in which they logged that they completed exercise) in the week following compared with the week preceding a prompt [32]. Self-monitoring and self-reported exercise data from the mHealth platform were averaged over 1, 3, 5, and 7 days before and after a prompt for the first and second half of the 12-month follow-up phase and were compared using *t* tests. The impact of the prompt was strongest in the first half of the year, with no significant differences found in the second half of the year. In the first half of the year, self-monitoring significantly increased in the 3 days following a prompt (*P*<.001; *d*=0.60), and self-reported exercise significantly increased in the 3 (*P*<.001; *d*=0.37), 5 (*P*=.04; *d*=0.14), and 7 days (*P*=.02; *d*=0.15) following a prompt.

Together, these secondary analyses provide preliminary evidence that mHealth prompts *may* be useful within the SSBC context, and more comprehensive development of an mHealth-prompting (to be sent in the future via SMS text messaging to improve scalability) intervention is warranted.

##### SSBC Example: Consultation of Previous Literature

Once the potential utility of an mHealth-prompting intervention was identified, a scoping review was conducted to identify how existing DPPs develop mHealth-prompting interventions [33]. The results from this review highlight that mHealth prompts are typically delivered via SMS text messaging, followed by push notifications, and that they are generally well-received by participants. However, both the development of prompt content (ie, what BCTs are used and how theory influenced content) and delivery of prompts (eg, frequency, timing, and duration of prompting interventions) are often underreported, highlighting the need for structured development and rigorous evaluation of mHealth-prompting interventions before deployment.

In addition to conducting our own review, previous reviews assessing SMS text messaging interventions were also consulted. To date, many reviews have assessed the impact of SMS text messaging on health behavior change interventions [34-41], and meta-analyses have consistently shown that SMS text messaging interventions have a significant effect on hemoglobin A_1c_ among individuals with diabetes [39,40,42], weight loss [36,37], and physical activity [38,43]. It is widely accepted that behavior change interventions *should* be developed using theory, past evidence, and formative research [20,44] and that sufficient intervention detail be reported to allow for replication [45,46]. However, many SMS text messaging interventions lack rigorous development and thorough reporting, thereby limiting their utility in future intervention development and implementation [47].

In addition to how SMS text messaging interventions are developed, the description of theoretical mechanisms within message content and reporting of delivery characteristics (eg, timing and frequency) are largely unspecified in the literature [48]. With respect to SMS text messaging delivery, the evaluation of weight management SMS text messaging interventions by Skinner et al [36] included subgroup analyses examining intervention duration (<6 months vs 6 months vs 12 months), message frequency (daily vs weekly or biweekly vs personalized), and 1- versus 2-way messaging and found no significant subgroup differences. The authors noted that poor intervention descriptions within publications may have affected their ability to accurately code aspects of intervention delivery. Although a trend was noted that less frequent messaging (weekly or biweekly) was associated with greater reductions in weight (mean −2.88 kg, 95% CI −4.56 to −1.21 kg) than for daily messages (mean −1.56 kg, 95% CI −2.26 to −0.86 kg), the authors concluded that the mechanisms of action by which SMS text messaging programs lead to these effects remain largely unclear, and further investigation into message delivery and content features is warranted. This is mirrored by other reviews that note the heterogeneity in message timing, frequency, and duration within the existing literature and call for future research to determine the optimal message dose for health behavior change [35,49].

#### Step 3: Select Potential Intervention Components

##### Recommendation

The selection of intervention components should be performed early in the development process to identify different component levels. Component levels include the different doses of an intervention component one wishes to test (eg, high or low dose or turning a component on or off). By selecting components early, adequate time may be invested in preparatory development work to select appropriate component levels and consider how they will be tested. The detection of key intervention components should be based on theory, evidence, and end users [28,50,51].

Steps 1 and 2 may help to clarify intervention components that are either (1) based on existing evidence or (2) currently under researched and may be studied to move the field forward. In this way, evidence-based intervention components may be incorporated into the intervention package, and where evidence for a potential intervention component does not yet exist, researchers are able to further study the mHealth intervention component of interest (eg, if there is no existing evidence on how the frequency of intervention contact may influence a target population, but previous theory or evidence suggests that it should influence their behaviors, inclusion of that delivery component can allow a new line of evidence to be built for the target population).

The integration of previous research evidence and theory can provide insight into what intervention components have been previously identified as having the potential to be effective; however, unless these studies were conducted with the population of interest, they are unlikely to provide guidance on which intervention components are most important or can be best implemented within a given context [28]. To ground mHealth development within the implementation context, the Person-Based Approach suggests engaging in in-depth qualitative research [28]. Understanding user perspectives and accommodating their priorities within the candidate intervention components can aid in maximizing the acceptability and uptake of interventions when they are at the implementation stage [28].

##### SSBC Example

Based on qualitative work profiling SSBC participants, participants wanted continued support from their coach and additional information regarding prediabetes after the completion of the training phase, and they found mHealth technologies to be facilitators for maintaining their long-term behavior change [26,27]. From the results of the scoping review, the intervention components to be tested in the SSBC MOST optimization trial included SMS text messaging content and delivery components (ie, timing and frequency of prompt delivery) [33]. By understanding what message content SSBC participants would like to see (and the underlying theoretical mechanisms) and by optimizing SMS text messaging delivery to best facilitate behavior change, this research may reduce intervention costs and promote user engagement, ultimately reducing T2D risk.

#### Step 4: Place Constraints on mHealth Development to Improve Affordability, Scalability, and Efficiency

##### Recommendation

The MOST recommends that interventions be developed and optimized to meet resource-related implementation considerations (eg, constraints on personnel time, costs, or complexity of the selected technology). Typically, interventions are developed without considering affordability or the ability to be implemented as designed, which results in considerable research funds and time devoted to establishing efficacious interventions that are not practical in the real-world context [52]. By accounting for implementation constraints at the onset, the MOST aims to achieve intervention EASE by balancing the effectiveness of an intervention against relevant implementation constraints on affordability (eg, can be developed and delivered within certain budgetary constraints), scalability (eg, can be implemented with high fidelity), and efficiency (eg, contains only *active* intervention components).

By considering potential end users when designing mHealth content, researchers can tailor development and delivery based on the intended implementation context, thereby improving translation into practice [10]. Specifically, these constraints can help balance the intervention *EASE* while developing a specific optimization objective. Intervention optimization objectives can be broad (eg, achieving the best outcomes for the lowest price) or specific (eg, improving physical activity by a minimum of 15 minutes per day while keeping the overall intervention cost <US $300), and the level of specificity will depend on the constraints outlined at the onset and the requirements and resources of potential end users.

##### SSBC Example

When developing the SMS text messaging intervention, constraints were placed on the messages themselves, and the platform that was chosen to deliver the messages. The optimization objective was defined as identifying the SMS text messaging intervention that increases physical activity adherence most during the SSBC follow-up phase, given the identified constraints.

###### Text Messaging Constraints

Before message development, the following message constraints were put in place to improve the reach and scalability of the current intervention: messages must be written so that they can be sent as automated, 1-way messages (to reduce the burden on SSBC coaches), and they must be <160 characters long (to ensure they fit into a single SMS text message for individuals without a smartphone). These decisions were made so that the development process used in the current program of research could be adapted for any behavior change scientist or health care professional who may not have the resources necessary to create or invest in mHealth platforms that use advanced decision-making algorithms to provide just-in-time adaptive SMS text messaging interventions.

###### Text Messaging Platform Constraints

To reduce the person-hours required for sending messages and allow for fidelity assessment in future research, the following constraints were identified when selecting the SMS text messaging platform for use in this research program. The platform must be able to schedule and queue messages with rolling start dates; allow for variable timing, content, and frequency within scheduled messages; provide analytics such as audit logs of messages sent, declined, or undeliverable; and provide opt-out reports for participants requesting to unsubscribe from receiving messages.

#### Step 5: Develop mHealth Content

##### Recommendation

To improve the implementation of evidence-based mHealth research in real-world contexts, it has been suggested that intervention content should be developed (1) using a dynamic and iterative process including end users, (2) based on existing research evidence and theory, and (3) with the implementation context in mind (eg, clinical workflows) [28,53]. The integration of specific theoretical frameworks such as the Behaviour Change Wheel (BCW) has been suggested to systematically identify candidate intervention components and develop theory-based mHealth content that is underpinned by specific mechanisms of action (note that the use of such theoretical frameworks can also be helpful when developing a conceptual model) [21]. The BCW is a synthesis of 33 behavior change theories that collate the barriers and facilitators needed to change a target behavior.

##### SSBC Example

The development and refinement of SMS text messaging content for SSBC were split into 3 parts: identification of BCTs, message development, and message evaluation and refinement.

###### Identification of BCTs

To determine which BCTs were already in use within the SSBC program, 2 coders trained in BCT identification assessed all SSBC program materials and standard operating procedures [54] using the systematically developed taxonomy of BCTs (BCT Taxonomy version 1) [55]. BCTs are the building blocks of an intervention and are defined as the smallest *content components* within an intervention. Depending on the granularity in which a researcher wishes to assess the intervention components, BCTs can be toggled on or off for a more rigorous assessment during the optimization phase of the MOST.

###### Message Development

**Figure 1:**
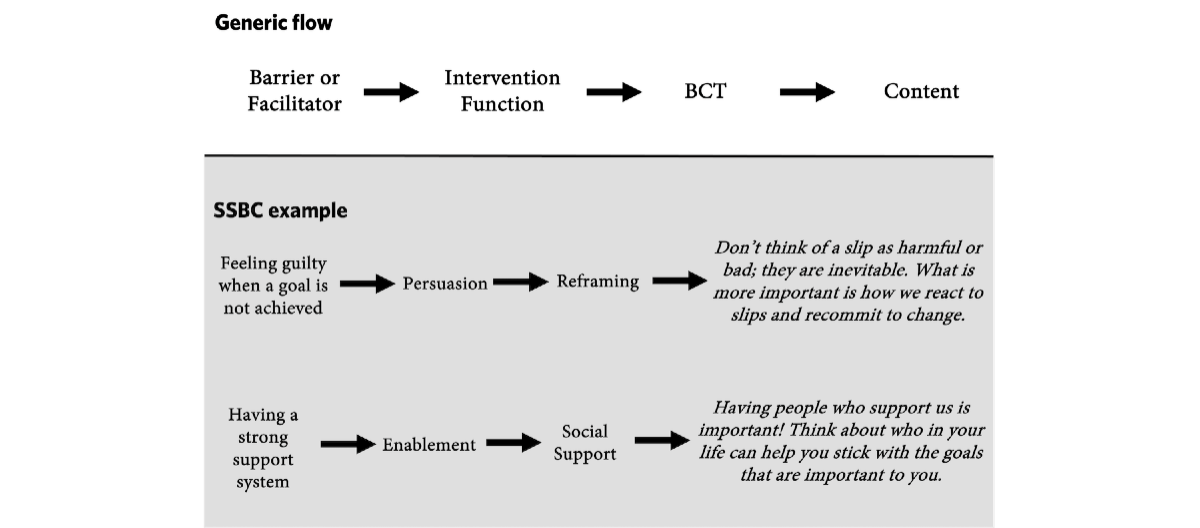
Theoretical content development using the Behavior Change Wheel. BCT: behavior change technique; SSBC: Small Steps for Big Change.

The BCW [56] was used to develop a bank of messages [57] linking the relevant BCTs previously identified with theoretical mechanisms by which the messages should influence behaviors. Previous qualitative research profiling SSBC participant journeys in the year following the program [27] was used to identify barriers to and facilitators of maintaining dietary and physical activity behaviors after program completion. These barriers and facilitators were then linked to intended intervention functions (eg, education, persuasion, and enablement) and previously identified BCTs to formulate a bank of 124 theory-based messages based on participant-identified barriers and facilitators (Figure 1). Decisions on which intervention functions and BCTs to use in the messages were guided by the *APEASE criteria*, which may aid in achieving intervention EASE. APEASE represents affordability, practicability, effectiveness, acceptability, side effects/safety, and equity [56]. By critically appraising each intervention function and potential BCT using the APEASE criteria, only those components likely to elicit changes that are also likely to be implemented in the given context may be included in the development of mHealth content.

Messages were written to target diet or physical activity behaviors or provide more general motivation and education; this was done by tailoring content to target client–identified barriers or facilitators toward improving client confidence in their ability to maintain the diet and physical activity changes made during the SSBC training phase.

###### Message Evaluation and Refinement

After development using the BCW, key knowledge users (SSBC coaches and past SSBC clients) evaluated the messages on their readability, relevance, and utility for individuals at risk of developing T2D [58]. Overall, messages were rated highly by both coaches and participants (receiving an average score of 13.77 out of a possible 15). General motivational messages (eg, “Your first plan will not work 100% of the time. Continue to change your goals until you find what works best for you*!*”) were generally scored among the highest compared with targeted behavioral messages (eg, “Think about where, when and how you’ll get your exercise in today!”). In addition, while evaluating messages, participants were asked if they were to send or receive messages similar to those they evaluated, how many they would like to send or receive weekly, and for how many months. SSBC coaches reported that they would like to send an average of 3 messages per week (mode 3, range 1-5) for 5 months (mode 1, range 1-12), and past SSBC clients reported that they would want to receive an average of 3 messages per week (mode 2, range 2-5) for 7 months (mode 12, range 2-12).

#### Step 6: Identify Intervention Component Levels

##### Recommendation

Where previous experimental evidence exists, as identified in the review of existing literature (step 2), it should be used to inform intervention component levels, warranting further testing. If no experimental evidence exists for different component levels or the objective is to further the science of mHealth development and evaluation, different components or component levels may be chosen. At this stage, intervention developers should begin to think about the study design for future intervention optimization trials; these can include factorial, sequential multiple assignment randomized trials (SMART) and microrandomized trials to name a few. The design of the optimization trial is driven by the research questions and available resources. When conducting a factorial experiment, the most efficient way (and the one recommended by the MOST) is to stick with 2 levels for each component (eg, on or off or high or low dose). Once the MOST optimization trial design has been decided, researchers can consider the candidate intervention component levels to be tested. Component levels can be as broad or as specific as needed and often depend on the nature of the intervention and research questions.

##### SSBC Example

###### Overview

**Figure 2:**
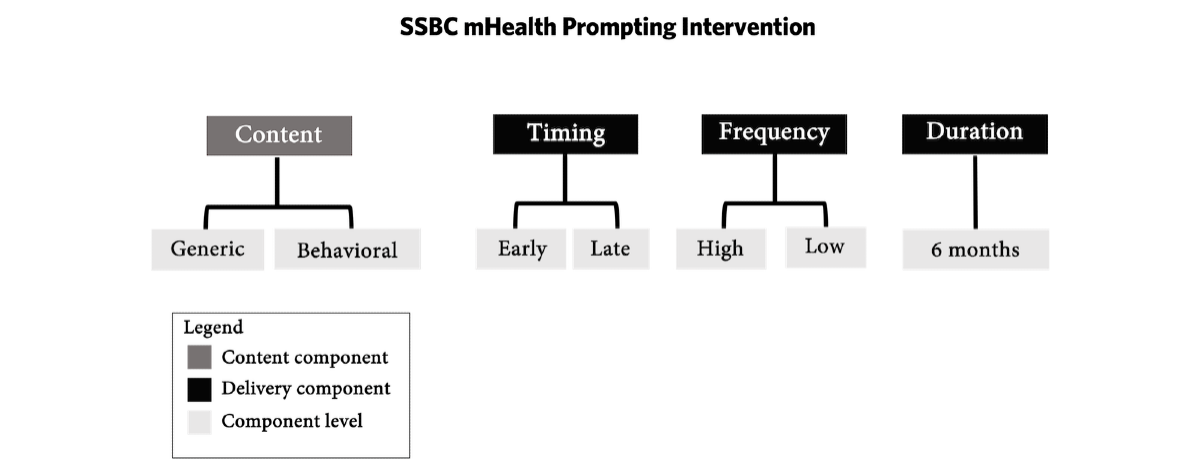
Intervention components and component levels. mHealth: mobile health; SSBC: Small Steps for Big Change.

Our previously identified intervention components included *prompt content* and *message delivery* (ie, duration, timing, and frequency; Figure 2). As this intervention is intended to be implemented by SSBC coaches and potentially other health care practitioners, a factorial experiment with 2 levels for each component was chosen. Although it is likely that the *optimal* (in terms of effectiveness) prompting frequency may change over time and be specific to the client receiving the prompts (thus lending itself better to a SMART or microrandomized trial), health care and public health services are unlikely to have the resources available to tailor prompting delivery to each individual client, making a standard delivery frequency more likely to be implemented into practice.

###### Prompt Content

As each message was carefully curated and linked to specific BCTs, it would theoretically be possible to test different BCT categories or compare those messages that include one versus multiple BCTs; however, based on participant feedback in the evaluation and refinement survey, we will test 2 levels in our future optimization trial: general and targeted behavior content.

###### Message Delivery

Based on secondary analyses showing that prompts were most effective in the first 6 months following the SSBC training phase, coupled with diabetes prevention coaches and past SSBC clients wanting to receive messages for approximately 6 months, we decided not to include another level for this component, thereby defaulting the intervention *duration* to 6 months for all participants. In addition, our review highlighted that prompt *timing* is grossly underreported in diabetes prevention programming; thus, we chose to set the delivery component to *early* or *late* in the day. To identify a specific timing, participant preferences for timing (morning, afternoon, or evening) are being solicited in an ongoing feasibility study (further discussed in the following sections) [59].

For prompt *frequency*, our previous research (ie, secondary analyses assessing the acute impact of a prompt and message evaluation survey) [31,32] suggests that more frequent messages (eg, 6 or 7 per day) are burdensome and are therefore unlikely to positively affect the target behaviors. In addition, none of the past SSBC clients reported wanting only a single message within a week. On the basis of the paucity of data on optimal prompting frequency coupled with these secondary analyses and participant preferences, a frequency between 2 and 5 messages per week is suggested to optimally affect self-monitoring and self-reported exercise. Therefore, we chose to make the weekly prompt frequency a *high* or *low* dose. Again, the specific dose will be identified after the completion of the ongoing feasibility study.

#### Step 7: Develop a Conceptual Model

##### Recommendation

Creating a conceptual model to link intervention components to their theoretical mediators and expected outcomes may improve the ability of an intervention package to be effectively adapted to different contexts or settings [60]. Developing a conceptual model within the preparation phase of the MOST can be completed using a number of potential theories. As pinpointing a specific theory that fits one’s needs can be overwhelming, the use of a theoretical framework such as the BCW can aid mHealth researchers in developing a conceptual framework based on theoretical constructs [21].

##### SSBC Example

**Figure 3:**
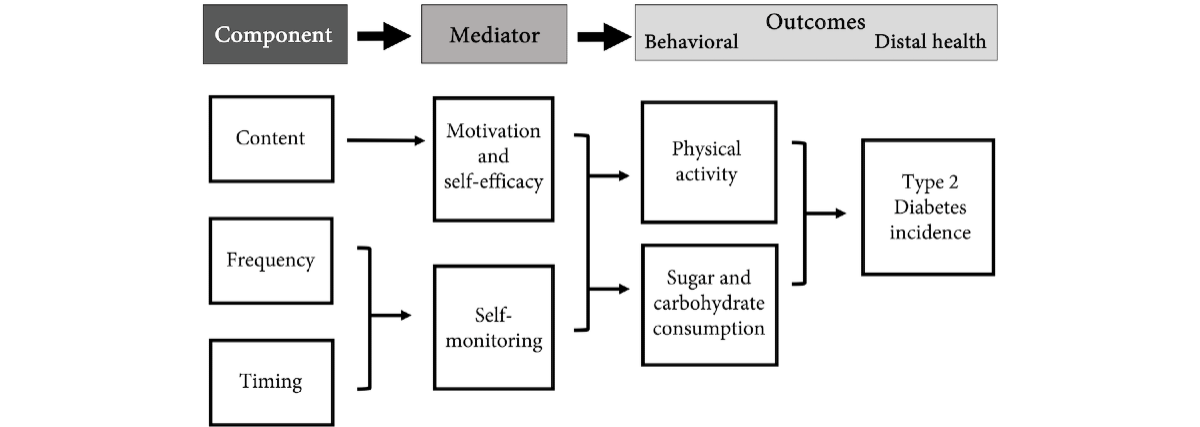
Conceptual model.

Based on the identified intervention components of prompt content and message delivery (timing and frequency), a conceptual model was created (Figure 3) to outline how these components are anticipated to influence target behaviors (dietary and physical activity behavior change adherence). Although each individual message is made up of different BCTs targeting different barriers and facilitators, the content generally aims to increase an individual’s motivation and self-efficacy to adhere to the diet- and exercise-related behavior changes made during the SSBC training phase. From our secondary analyses, it appears that the prompting dose may influence a participant’s behaviors by encouraging them to continue to self-monitor their behaviors.

#### Step 8: Run a Feasibility Study

##### Recommendation

Before the optimization phase, Collins et al [20] suggest conducting a feasibility study to establish whether each candidate component and component level can be feasibly implemented as intended. Feasibility studies are a common step in the process of developing and translating social science and public health interventions. Conducting a feasibility study can provide necessary information on participant recruitment, intervention acceptability, and the feasibility of administering an intervention as intended. This information may be used to aid in the decision to (or not to) conduct a fully powered optimization and efficacy trial. Furthermore, this may ensure that resources are not wasted in conducting an optimization trial if the intervention itself cannot be delivered as intended [61]. The primary aim of a feasibility study should be to inform the feasibility or acceptability of an intervention and identify modifications that need to be included within the intervention before a large-scale trial. As feasibility studies are not fully powered trials, conducting inferential statistical tests is discouraged, as *P* values rely on sample size [61]. If a researcher needs to make decisions about the inclusion or exclusion of an intervention component or component level, they should identify a priori how they intend to use the feasibility study data to inform this decision making. It is recommended that participant acceptability and preferences be used instead of *P* values in addition to effect sizes (which are less reliant on sample size).

##### SSBC Example

To test the feasibility and acceptability of the message delivery platform before optimization, a feasibility study is currently being conducted [59]. In addition to general intervention feasibility (ie, can it be delivered as intended), this study aims to assess participant preferences regarding message timing and frequency to further refine intervention levels. The feasibility study will result in a final set of mHealth-prompting delivery characteristics for further testing in the second phase of the MOST, using a factorial experiment.

## Discussion

Given their considerable reach, mHealth interventions have the potential to positively affect public health by decreasing implementation costs and improving accessibility. The impact can be maximized through rigorous development followed by optimization to ensure that candidate mHealth interventions meet the real-world contexts they seek to serve. Transparent reporting should also be prioritized to promote replicability and use beyond the intended scope, where applicable. In addition, these steps may promote intervention packages that are cost-efficient and effective without including unnecessary or potentially detrimental intervention components that could reduce the overall potency of the intervention. The MOST provides an example of a framework suitable for such development. The MOST follows 3 phases to identify components, pinpoint optimal delivery, and evaluate the efficacy of a final intervention package.

This paper provides an example of how the MOST was used to develop an mHealth-prompting intervention for the SSBC program situated within the preparation phase of the MOST. In addition, although this paper may serve as a guide for future mHealth researchers to develop mHealth interventions using the MOST, some of these steps (or the order in which they have been presented) may not be applicable for all mHealth development. Consequently, researchers should adjust their approaches to meet their own contextual needs. Despite the applicability of each individual step, mHealth development using the MOST should consider integrating concepts from agile innovation, the Person-Based Approach, and user-centered design to improve the likelihood that their mHealth development is grounded in the context of those who will be using the intervention and therefore is more likely to be integrated into routine clinical practice following evaluation [10,28,29].

## Appendix

Multimedia Appendix 1 The MOST PREP-REP (Multiphase Optimization Strategy Preparation Reporting) checklist.

## Abbreviations

APEASE: affordability, practicability, effectiveness, acceptability, side effects/safety, and equity
BCT: behavior change technique
BCW: Behaviour Change Wheel
DPP: diabetes prevention program
EASE: effectiveness, affordability, scalability, and efficiency
mHealth: mobile health
MOST: Multiphase Optimization Strategy
PREP-REP: Preparation Reporting
RCT: randomized controlled trial
SSBC: Small Steps for Big Change
T2D: type 2 diabetes
