# Supplementary material for: Developing Mobile Health Interventions With Implementation in Mind: Application of the Multiphase Optimization Strategy (MOST) Preparation Phase to Diabetes Prevention Programming
Source: JMIR Form Res. 2022 Apr 26;6(4):e36143. doi: 10.2196/36143 (PMC9092234; doi:10.2196/36143)
Supplement: Multimedia Appendix 1 [file formative_v6i4e36143_app1.pdf]

Fig 2 | The MOST PREP-REP checklist. MOST Multiphase Optimization STrategy; PREP-REP PREParation REPorting.

**Multiphase Optimization STrategy (MOST) PREParation REPorting (PREP-REP) Checklist**<br>This checklist should be used to guide the reporting of the process and outcomes of the MOST preparation phase in publications, presentations, and other research products.	**AUTHOR RESPONSES**
**1 Introduce The MOST Framework**<br>1.1 Identify the use of the multiphase optimization strategy (MOST) with appropriate citation(s)<br>1.2 Label aspects of current or prior work that the research team considers preparation for an optimization phase as the preparation phase	**1.1** MOST sub-section within the background.<br>**1.2** The entire publication is identified as preparation phase.
**2 Describe the Research Series**<br>2.1 Provide context for the current work in the introduction or background and clearly label prior, ongoing, or planned work as preparation where appropriate<br>2.2 Describe the trajectory of the research program leading up to the current work<br>2.2.a. Provide citations for any prior or associated work whether it was completed by the research team or by others (e.g., literature reviews, formative research, pilot studies, trials)<br>2.2.b. Connect the trajectory of the research program to the MOST phase(s) by labeling prior and current work with the appropriate phase (e.g., preparation phase, optimization phase) including any iterative movement between phases<br>2.2.c If space is available, provide example(s) of how prior work was translated into elements of the conceptual model and/or the optimization objective	**2.1** The context for the current work is within the Small Steps for Big Changes program described in the background section.<br>**2.2a** References 26, 27, 31-33, 54, 56, 58, 59 make up the preparation phase.<br>**2.2b** The entire publication is labeled as the preparation phase.<br>**2.2c** Conceptual model is described in Step 7 and Figure 3.
**3 Clearly Define the Conceptual Model**<br>3.1 Use the term conceptual model to identify the theory or framework guiding the selection of the components, mediators, and outcomes for optimization<br>3.2 Provide a detailed description of the conceptual model including a figure or diagram<br>3.2.a Identify and describe the intervention components and levels<br>3.2.b. Identify primary (and secondary) outcomes being targeted by the intervention components including descriptions of assessment tools<br>3.2.c Describe how each intervention component is purported to affect each outcome including descriptions of how relevant mediators will be assessed	**3.1** Conceptual model is described in Step 7 and Figure 3.<br>**3.2a** Step 6 and Figure 2 highlight components and component levels.<br>**3.2b** Primary and secondary outcomes are outlined in Figure 3.<br>**3.2c** Mediators are included in Figure 3.
**4 Optimization Objective**<br>4.1 Use the term optimization objective to identify the factors that will guide the selection of intervention components after the optimization trial<br>4.2 Provide an operational definition of the optimization objective<br>4.2.a Include specific criteria, if known, for determining which components will be retained in optimized intervention such as benchmarks related to effectiveness, affordability, scalability, and/or efficiency.<br>4.3 Describe the rationale for the selection of the optimization objective	**4.1** Optimization objective is described in Step 4.<br>**4.2** Our optimization objective is described in the *SSBC Example* in Step 4.<br>**4.3** Rationale for our optimization objective is discussed within the *SSBC Example* in Step 4

Transl Behav Med, ibab146, https://doi.org/10.1093/tbm/ibab146

The content of this slide may be subject to copyright: please see the slide notes for details.
